# Reply to Horacek, M. The Need to Consider Geochemistry When Interpreting Sr-Isotopes. Comment on “Gregorčič et al. The Provenance of Slovenian Milk Using ^87^Sr/^86^Sr Isotope Ratios. *Foods* 2021, *10*, 1729”

**DOI:** 10.3390/foods11040581

**Published:** 2022-02-17

**Authors:** Staša Hamzić Gregorčič, Nives Ogrinc, Russell Frew, Marijan Nečemer, Lidija Strojnik, Tea Zuliani

**Affiliations:** 1 Department of Environmental Sciences, Jožef Stefan Institute, Jamova 39, 1000 Ljubljana, Slovenia; stasa.gregorcic@ijs.si (S.H.G.); nives.ogrinc@ijs.si (N.O.); lidija.strojnik@ijs.si (L.S.); 2 Jožef Stefan International Postgraduate School, Jamova 39, 1000 Ljubljana, Slovenia; marijan.necemer@ijs.si; 3 Department of Chemistry, University of Otago, P.O. Box 56, Dunedin 9016, New Zealand; russell.frew@otago.ac.nz

We appreciate Dr Horacek’s interest in our paper and his feedback [1]. Indeed, we feel that we have already addressed his comments in our paper. For example, we are aware that the bedrock type has a greater influence on the ^87^Sr/^86^Sr ratios in soil than the age of the bedrock. However, our data agree with rock type and age (see [2] p. 8). For example, we write that the *“relationship between ^87^Sr/^86^Sr ratios in the milk samples and rock type at each sampling location was also explored”.* We also state how rock types and ages were obtained from the geological map provided by the Geological Survey of Slovenia [3]. Additionally, we point out that the ^87^Sr/^86^Sr isotope ratios in the milk samples agree with predicted values for Slovenia, as determined by Hoogewerff et al. [4], and refer to rock type, for example, when we write, *“…this information is in line with the bedrock composition and age”* and *“…most of the Slovenian territory is covered by tertiary and quaternary dolomites, limestones and alluvial deposits such as sandstones and claystones”.* Our statistical analysis reveals the differences and similarities between the rock types (Figure 6 in the original paper), so in the end, little disagreement exists between Dr Horacek’s comment and our paper.

We also go on to state how there “is a slight difference between milk samples from locations with quaternary alluvial deposits with alumo-silicate rocks with ^87^Sr/^86^Sr ratios ranging between 0.710 and 0.712, and locations with limestone and dolomite bedrock with ^87^Sr/^86^Sr ratios in the range from 0.708 to 0.710”. However, because of Slovenia’s complex geology, we decided to present the data and statistical evaluation using the age of the bedrock since, in this way, it allowed us to differentiate alpine carbonate from coastal carbonate areas. In addition, since only samples from soils above quaternary rocks differ significantly from other samples (*p* < 0.0001), we left this group separate. Furthermore, Dr Horacek is right in his assessment that statistical analysis based on the strontium ratio alone would not distinguish between the groups identified in the original paper, apart perhaps from the quaternary samples in the northeastern part of Slovenia. However, that is exactly what the statistical analysis in the original paper shows.

Regarding the two trends observed in Figure 4 [2] (Figure 3 in the Comment [1]), we explain their relevance as follows: *“Although several samples overlap, two trends can be identified: the first with high Sr concentration and high ^87^Sr/^86^Sr ratios (>0.7110) mainly from areas with quaternary alluvial deposits with alumo-silicate rocks and the second one related to lower ^87^Sr/^86^Sr ratios (<0.7090) in carbonate dominated areas*”. Perhaps a more extensive explanation would be that the first trend represents a mixing line from marine carbonate with low ^87^Sr/^86^Sr towards high ^87^Sr/^86^Sr influenced by siliciclastics, in the present case most probably coming from areas with Precambrian and Palaeozoic metamorphic and igneous bedrock (north-eastern Slovenia). In contrast, the second trend indicates different amounts of marine Sr in the carbonate bedrock.

As I am sure Dr. Horacek would agree, the relationship between strontium in the rivers and strontium in milk is complex. In our paper, we did not explicitly discuss the higher ^87^Sr/^86^Sr values in milk with respect to water but only pointed out the difference that has been noticed. However, we want to stress that the data could not be explained based only on geology, but many factors, such as farming practice in the area, are important. Indeed, additional explanation can be added to support our statements with respect to the relationship between elevated ^87^Sr/^86^Sr values in milk concerning ambient river water as follows: It is known that the addition of agricultural lime to the soil will lower the strontium ratio of the soil and the river water as the readily dissolvable agricultural lime will dominate runoff from the fields. However, suppose the lime is primarily applied to a field with cash crops for the largest return of investment to the farmer, and not to the hay field and grassland where the cows obtain the majority of their food. In that case, one could imagine a situation where strontium in the surface environment is very heterogeneously distributed, with higher ratios in the milk (less affected by agricultural lime) than in the rivers (more affected by agricultural lime). Thus, besides geology, detailed knowledge of land use is also important.

Finally, like Dr Horacek, we believe that ^87^Sr/^86^Sr ratios can be a powerful tool for determining the geographical origin of food originating from countries with more homogenous geology, while any interpretation based on ^87^Sr/^86^Sr ratios can be challenging for countries with heterogeneous geology, such as Slovenia and many other EU countries. This complexity is why we believe that geographical origin determination can be more powerful if ^87^Sr/^86^Sr data are combined with stable isotopes of light elements and elemental composition, which is the main conclusion of our paper.
